# Case report: Endovascular treatment of transplant renal artery stenosis in patient with normal color duplex ultrasound of the renal artery

**DOI:** 10.1002/ccr3.8492

**Published:** 2024-02-07

**Authors:** Javad Jalili, Hamid Tayebi Khosroshahi, Mehran Malekshoar, Mahshid Dehghan, Aisan Akhgari, Amirhosein Ghafouri Asbagh

**Affiliations:** ^1^ Department of Radiology Tabriz University of Medical Sciences Tabriz Iran; ^2^ Biotechnology Research Center Tabriz University of Medical Science Tabriz Iran; ^3^ Department of Radiology, Faculty of Medicine, Masih Daneshvari Hospital Shahid Beheshti University of Medical Sciences Tehran Iran; ^4^ Student Research Committee Tabriz University of Medical Sciences Tabriz Iran; ^5^ Cardiovascular Research Center Tabriz University of Medical Sciences Tabriz Iran

**Keywords:** case report, end‐stage renal disease (ESRD), percutaneous transluminal angioplasty, transplant outcomes, transplant renal artery stenosis

## Abstract

**Key Clinical Message:**

Proper diagnosis and treatment of vascular stenosis which is a possible complication of renal transplant is important in improving patients' quality of life and prognosis.

**Abstract:**

One known consequence among recipients of renal transplants is graft renal artery stenosis. Early identification and therapy are crucial to avoid graft malfunction and the serious consequences that might arise due to elevated hypertension in several organs. We report a rare case of transplant renal artery stenosis in a mid‐aged woman who presented with edema, hypertension, and increased creatinine 2 months after kidney transplant. The patient had normal renal arterial resistive index (RI) and perfusion index (PI), and there was only a modest decrease in perfusion on duplex ultrasound. Following the patient's renal stenting treatment, angiographic resolution was observed. After 14 days of regulated blood pressure following renal artery stenting, she was discharged from the hospital with her edema resolved. Considering complications in patients with clinical manifestations such as hypertension resistant to treatment and graft dysfunction, vascular stenosis is a notable issue to consider even in the context of normal renal arterial RI, PI, and duplex ultrasound. Proper diagnosis and treatment are of importance to improve patients' quality of life and prognosis.

## 1 BACKGROUND

Renal transplantation is the preferred course of treatment for most patients with end‐stage renal disease because it increases both survival and quality of life.^1^ One known consequence among recipients of renal transplants is graft renal artery stenosis. Clinical manifestations include graft malfunction, rising creatinine levels, and recent onset or refractory hypertension are most frequently observed.^2^ Therefore, it is crucial to establish early diagnosis and therapy to prevent graft malfunction and the serious consequences that may follow in various organs as a result of severe hypertension.^3^

To identify vascular issues as soon as feasible, subsequent examinations after kidney transplantation should be carried out, including studies to detect graft perfusion impairment. In this article, we report a rare case of transplant renal artery stenosis with a normal resistive index (RI) and perfusion index (PI) and only mildly decreased perfusion in duplex ultrasound, in a middle‐aged woman presenting with edema 1 month following renal transplant.

## 2 CASE PRESENTATION

**FIGURE 1:**
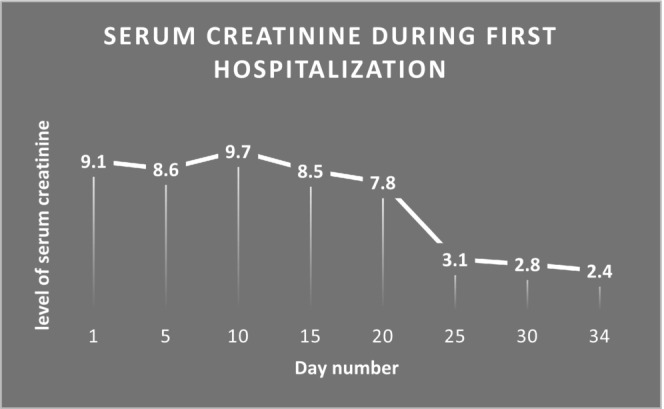
Serum creatinine trend during posttransplantation hospitalization.

A cadaveric kidney transplant was performed on a 56‐year‐old woman who had end‐stage renal failure from type 2 diabetes and a long history of hypertension, ischemic heart disease (IHD), and dyslipidemia. Before the transplant, the patient received hemodialysis treatment for 18 months. The donor was a healthy 50‐year‐old man who had sustained brain death. He had no history of urinary tract disease. By conventional procedure, the renal vein was anastomosed to the right external iliac vein and the renal artery to the right internal iliac artery. Due to delayed graft function (DGF), the patient's postoperative course was difficult and necessitated intermittent hemodialysis. During hospitalization, the patient underwent biopsy and it showed tubular injury with no rejection features. Twenty days after the procedure, prompt diuresis happened, and kidney function gradually became better. Urinary output reached 1.5–2 mL/kg/h, resulting in a significant creatinine serum level decrease (Figure 1). The patient was under observation for 10 more days and was placed on an immune suppression induction regimen with mycophenolic acid, tacrolimus, and prednisone. The tacrolimus level was between 12 and 15 ng/mL early posttransplantation and it was 10 ng/mL at the time of discharge. She was discharged on posttransplantation day 34 with a serum creatinine level of 2.4 mg/dL. Her blood pressure was controlled with metoprolol 50 mg daily and furosemide 40 mg daily. The patient's renal function and blood pressure remained stable over the next month.

**FIGURE 2:**
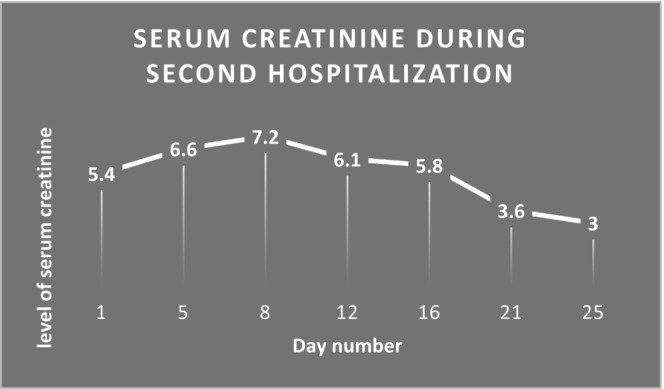
Serum creatinine trend during hospitalization due to posttransplantation renal artery stenosis.

One‐month after discharge, she was admitted to the hospital with a chief complaint of peripheral edema. Physical examination and laboratory assessments showed elevated blood pressure, serum creatinine levels of more than 5 mg/dL, and a decrease in urine output (Figure 2). Table 1 summarizing laboratory data at the time of admission is shown in below.

**TABLE 1:** Laboratory findings at the time of admission. (Normal ranges are shown in parentheses).

WBC: 13.4 (4–11)	MCV: 81 (80–98)	PLT: 296,000 (150–450 × 1000)	K: 5.6 (3.7–5.5)	Total bilirubin: 0.5 (0.2–1.2)	Calcium‐total: 7.6 (8.6–10.3)
RBC: 3.73 (4–5.2)	MCH: 22 (27–32)	Urea: 128 (15–40)	AST: 12 (0–37)	Direct bilirubin: 0.2 (0–0.4)	Calcium‐ionized: 1.04 (1.13–1.30)
HB: 10.6 (12–16)	MCHC: 27 (32–36)	Creatinine: 5.4 (0.7–1.4)	ALT: 13 (0–31)	Indirect bilirubin: 0.30 (0.1–0.8)	Phosphorus: 4.8 (2.5–5)
HCT: 32.5 (36–48)	RDW: 15.4 (11.5–15)	Na: 135 (136–145)	Alk.p: 124 (64–306)	Blood sugar: 162 (70–140)	Mg: 1.5 (1.7–26)

Abbreviations: Alk.p, alkaline phosphatase; ALT, alanine transaminase; AST, aspartate aminotransferase; HB, hemoglobin; HCT, hematocrit; K, potassium; MCH, mean corpuscular hemoglobin; MCHC, mean corpuscular hemoglobin concentration; MCV, mean corpuscular volume; Na, sodium; PLT, platelet; RBC, red blood cell; RDW, red cell distribution width; WBC, white blood cell.

During hospitalization, the patient's creatinine increased to 5–7 mg/dL, and her hypertension became more difficult to control. Due to a low glomerular filtration rate (GFR) and progressive edema, the patient underwent several hemodialyses.

## 3 METHODS

**FIGURE 3:**
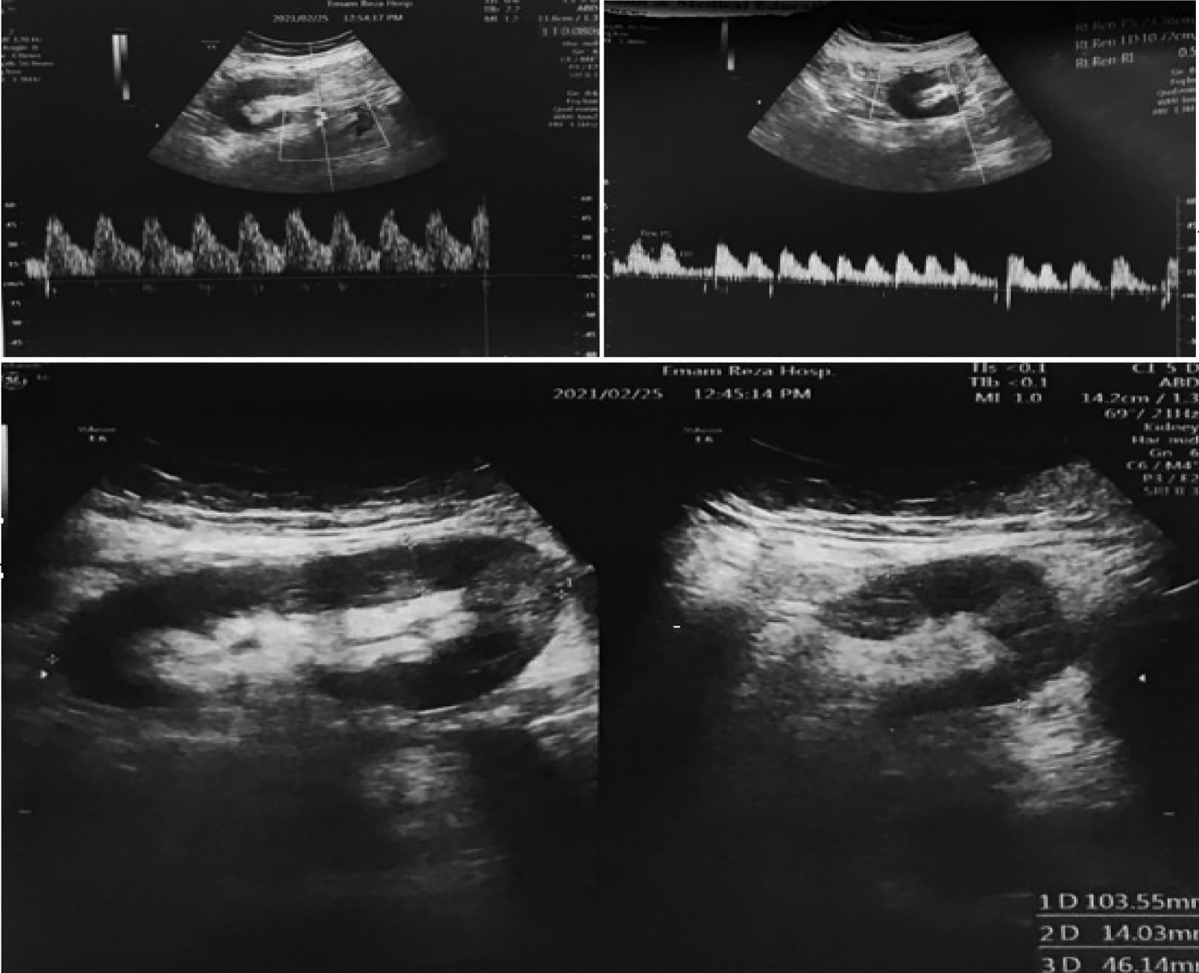
Duplex ultrasound of the patient; showing decreased vascular perfusion, there is no signs of artery or vein stenosis of the renal transplant.

**FIGURE 4:**
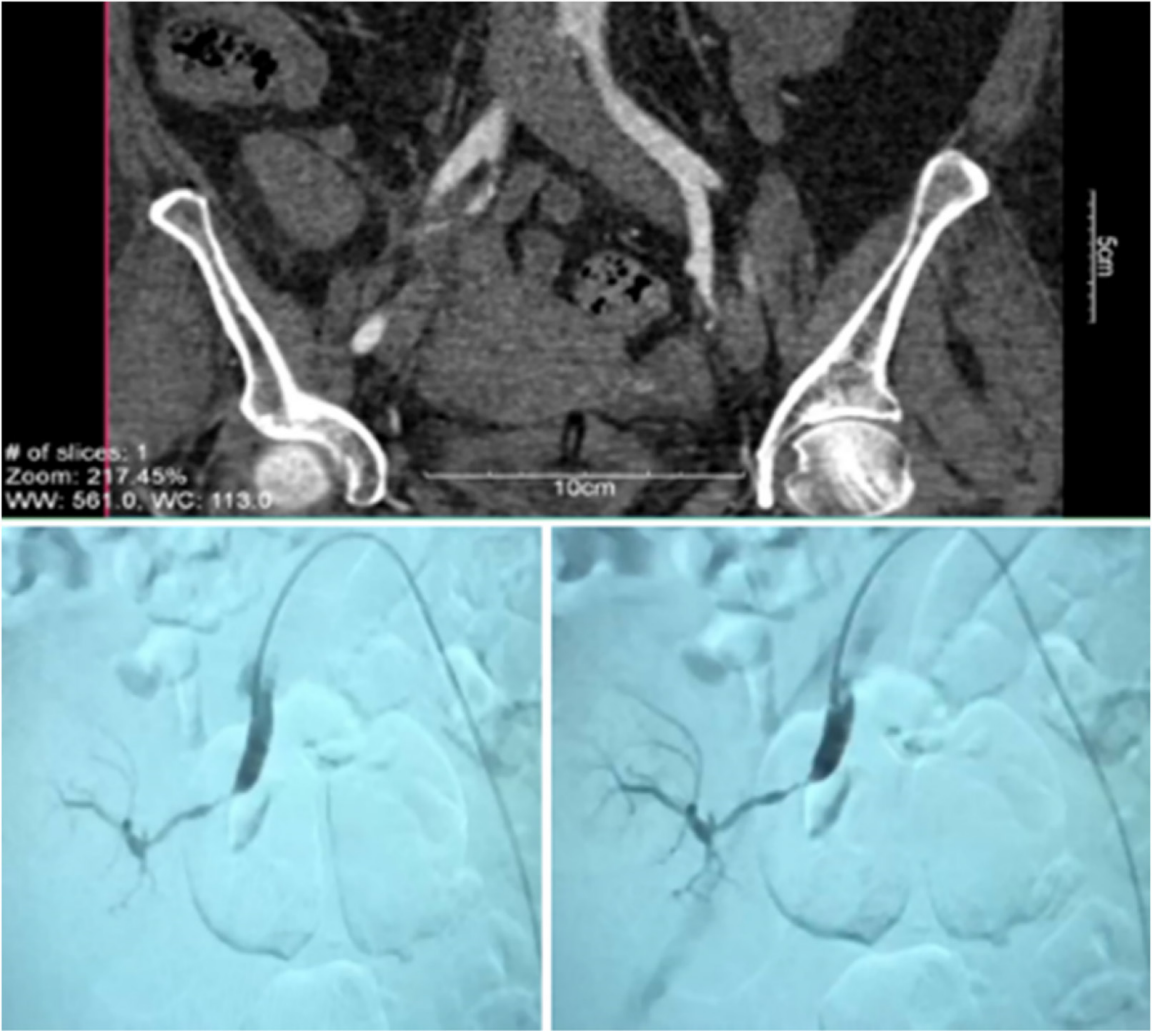
CT angiography of abdomen and pelvis and renal angiography showing an 80% or greater stenosis of the origin of the transplant renal artery.

A duplex ultrasound of the renal transplant showed mildly decreased vascular perfusion with a normal renal arterial RI of 0.60. No fluid collection or hydronephrosis was seen in the duplex ultrasound. To identify the reason for the patient's low GFR, two more duplex ultrasounds were performed. Except mildly decreased vascular perfusion, no signs of artery or vein stenosis of the transplant were seen in any of the performed duplex ultrasounds (Figure 3). The patient was examined through other imaging techniques as the presence of stenosis was suspected. Computed tomography angiography (CTA) of the abdomen and pelvis confirmed an 80% stenosis of the origin of the transplant renal artery (Figure 4). Five days before the procedure aspirin 80 mg and clopidogrel 75 mg were administrated. Renal artery stenting was performed by administering intravenous fixed‐bolus (5000 units) unfractionated heparin at the beginning of the procedure. The procedure was performed with a small amount of an iodinated contrast agent. A 5FR introducer sheath (Guider Softip, Stryker) 11 cm long was placed at the left common femoral artery. After that, the guiding catheter was inserted into the right iliac artery. Angiography showed significant stenosis (80%) at the origin of the transplant renal artery (anastomotic segment). After passing the guidewire (0.014, BMW, Abott vascular) through the stenotic segment, stenosis was treated by a balloon‐mounted drug‐eluting stent (4.00 × 12, Promus Premier, Boston Scientific). This interventional procedure took <40 min.

## 4 RESULTS

**FIGURE 5:**
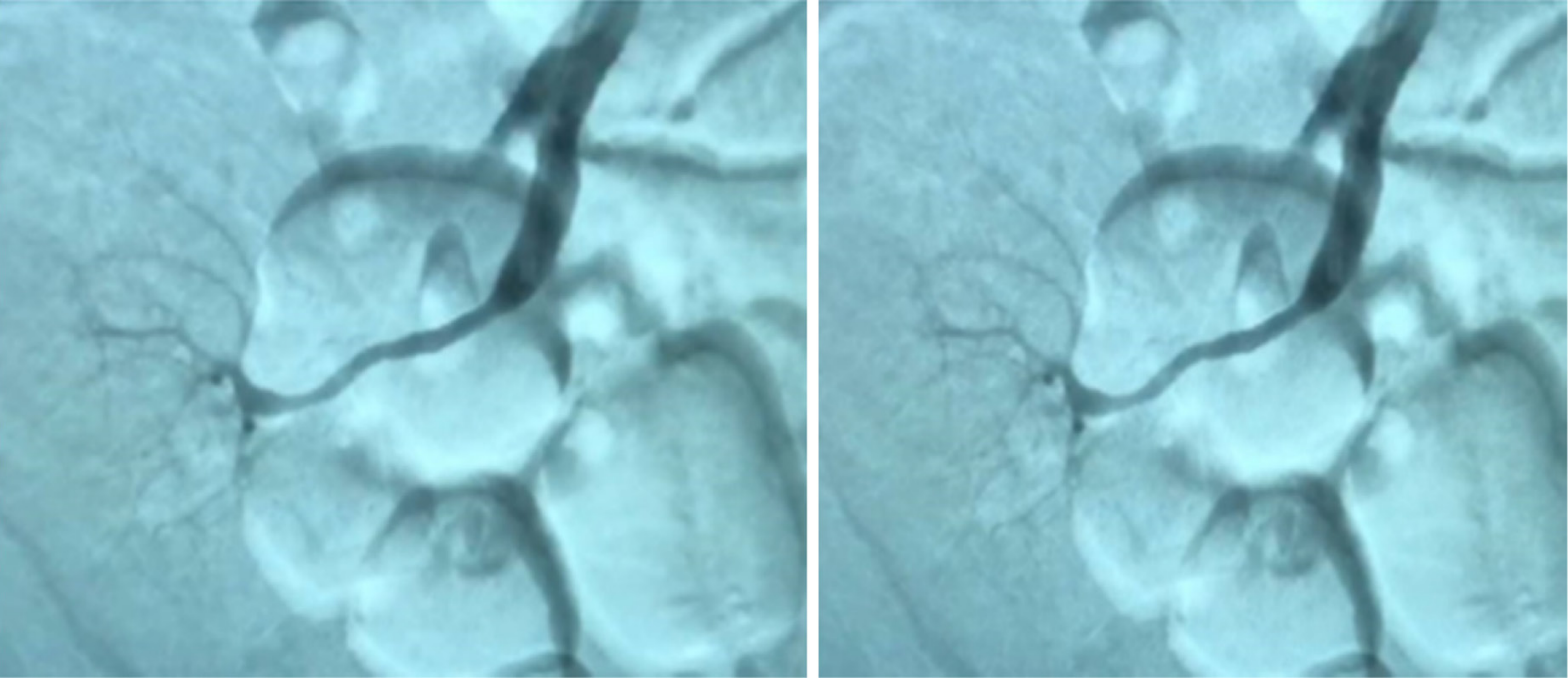
Post stenting renal angiography of the patient; showing angiographic improvement of the lesion with no residual arterial pressure gradient across the anastomosis.

Renal artery stenting resulted in angiographic resolution of the lesion and no residual arterial pressure gradient was seen across the anastomosis (Figure 5). Ten days following renal artery stenting, the patient's GFR significantly improved and his urine output gradually increased. As a result, the patient's edoema subsided and two antihypertensive drugs were used to maintain good blood pressure management. Aspirin 80 mg and Plavix 75 mg one tablet per day started 5 days before angioplasty and stenting and continued until 6 months after the procedure and then Plavix was discontinued. After renal artery stenting, the patient was discharged from the hospital 14 days later, with a blood creatinine level of 3 mg/dL and a normal renal duplex with RI = 0.61. Also the control duplex ultrasonography of the renal transplant revealed a normal transplant renal artery 1 month following the stenting. Serum creatinine levels were 3.41 mg/dL, and the patient experienced no more adverse effects.

## 5 DISCUSSION AND CONCLUSION

Vascular complications are common causes of renal transplant dysfunction, occurring in 15% of renal transplant patients and leading to high morbidity and mortality rates.^4^ These complications include renal artery or vein thrombosis, renal artery stenosis, arteriovenous fistulas, pseudoaneurysms of the renal artery, and finally, and less commonly, iliac artery stenosis.^5^, ^6^, ^7^, ^8^ The most common vascular complication is graft artery stenosis at the site of the anastomosis with the iliac artery of the recipient.^9^

Risk factors for the development of artery stenosis are assumed to be the same as for atherosclerotic disease including hypertension, diabetes, hyperlipidemia, smoking, and underlying atherosclerotic disease.^10^ Therefore, stenotic lesions commonly occur in transplant patients with underlying peripheral arterial disease and atherosclerotic damage to the vessels. Furthermore, suture technique and vascular clamp injury to the vessel lead to intimal hyperplasia and stenosis.^2^

Vascular complications present with worsening renal function, edema, severe hypertension (an increasing requirement for antihypertensive drugs) with or without graft dysfunction, elevated serum creatinine levels, bruits, and lower limb claudication. In patients with clinical manifestations of stenosis, biochemical workup and imaging studies of iliac and graft vessels such as duplex ultrasound, CTA, or magnetic resonance angiography (MRA) should be performed. Even though duplex ultrasound is highly sensitive, it is associated with a specificity of 75% and a positive predictive value of only 56%. As in this case, duplex ultrasound showed false‐negative results.

Stenting for artery stenosis is the first‐choice treatment that is widely accepted. Renal artery stenting effectively controls and improves hypertension and is generally less morbidity‐causing than surgery. In cases where renal artery stenting is not effective or when very severe stenoses prevent access, surgery may be necessary. The surgical intervention's optional approaches include patch grafting, stenotic segment saphenous vein bypass graft, localized endarterectomy, and anastomosis resection and revision.^2^ Stent migration and embolisation, side branch obstruction, late thrombosis, rupture, and aneurysm formation are uncommon complications associated with stent implementation.^8^, ^11^ In order to ensure optimum renal function and overall transplant success, posttransplantation renal artery stenosis (PTRAS) management is essential. To avoid worsening renal artery stenosis and preserve transplant viability, cautious medication selection is necessary in addition to interventional treatments like angioplasty or stenting. Fluid retention and renal vasoconstriction are two side effects of nonsteroidal anti‐inflammatory drugs (NSAIDs). These effects may worsen PTRAS in patients by raising blood pressure and decreasing renal blood flow.^12^

The renin‐angiotensin‐aldosterone pathway may be blocked by angiotensin‐converting enzyme inhibitors (ACEIs) and angiotensin II receptor blockers (ARBs). This could result in efferent arteriolar dilatation and elevated intraglomerular pressure. This might have a negative impact if renal artery stenosis is present. Volume loss caused by diuretics can trigger the renin‐angiotensin‐aldosterone pathway. Renal artery stenosis may worsen and vascular resistance rise as a result of this.^13^ Certain immunosuppressive drugs, such tacrolimus and cyclosporine, can constrict the blood vessels in the renal system.^14^ All in all these kind of medications may further impair blood flow to the transplanted kidney in PTRAS patients. It is critical to avoid some drugs, such as diuretics, ACEIs, ARBs, NSAIDs, and some immunosuppressants, in order to reduce the risk of renal artery stenosis getting worse and to support the long‐term success of kidney transplantation.

Andreas Skraeddergaard et al. described a 16‐year‐old girl with refractory HTN in a case report. This patient was finally diagnosed with renovascular HTN, and the definitive diagnosis was obtained via CTA. Only an expert can perform and interpret duplex ultrasound to aid in diagnosis. Renal artery stenosis can be difficult to diagnose, and in situations where the clinical symptoms are suggestive of renovascular hypertension but stenosis cannot be identified even with CTA, angiography may be required to rule out stenosis.^15^ Brooklyn L. DeVries and colleagues reported a transplant renal artery stenosis in a 67‐year‐old man in another study. In this case, duplex ultrasound revealed low‐normal vascular flow velocities, and a renal angiography was performed to confirm the patient's diagnosis. Finally, the patient recovered through surgical repair.^16^ In another case report study, Osama Safdar et al. observed that diagnosing renal artery stenosis might be difficult. The patient in that research completed several tests before undergoing percutaneous transluminal angioplasty as a diagnostic and therapeutic strategy.^17^

In summary, patients' quality of life and prognosis can be improved by taking into account the possibility of vascular stenosis, a potential renal transplant complication that can result in high rates of morbidity and mortality in recipients. This is particularly relevant for patients who demonstrate clinical manifestations like hypertension that is resistant to treatment and graft dysfunction. Due to the low occurrence of this transplant complication, further investigation is required to determine the true incidence and provide firm treatment guidelines.

## AUTHOR CONTRIBUTIONS

**Javad Jalili:** Conceptualization; supervision. **Hamid Tayebi Khosroshahi:** Methodology; supervision. **Mehran Malekshoar:** Conceptualization; supervision. **Mahshid Dehghan:** Writing – original draft. **Aisan Akhgari:** Writing – original draft. **Amirhosein Ghafouri Asbagh:** Writing – original draft; writing – review and editing.

## FUNDING INFORMATION

There was no funding for the research for this manuscript for either author.

## CONFLICT OF INTEREST STATEMENT

The authors declare that they have no competing interests.

## ETHICAL STATEMENT

Ethics committee of Tabriz University of Medical Sciences does not require approval and review for case reports.

## CONSENT

The authors have obtained the patient's written informed consent for print and electronic publication of this case report.

## Data Availability

Identifying patient data could not be shared. Most of the other clinical data used in this case report is presented in the manuscript. More detailed information is available from the corresponding author on reasonable request.

## References

[1] Spinosa DJ , Isaacs RB , Matsumoto AH , Angle JF , Hagspiel KD , Leung DA . Angiographic evaluation and treatment of transplant renal artery stenosis. Curr Opin Urol. 2001;11(2):197‐205. 11224752 10.1097/00042307-200103000-00012

[2] Mitema D , Schinstock C . Unique considerations when managing hypertension in the transplant patient. Hypertension: from basic research to clinical practice. Adv Exp Med Biol. 2016;956:341‐353. 10.1007/5584_2016_87 27815930

[3] Mannemuddhu S , Pekkucuksen N , Bush R , Johns F , Upadhyay K . Transplant renal artery stenosis in a child with BK nephropathy. Pediatr Transplant. 2020;24(1):e13629. 31815337 10.1111/petr.13629 PMC7167878

[4] Sagban TA , Baur B , Schelzig H , Grabitz K , Duran M . Vascular challenges in renal transplantation. Ann Transplant. 2014;19:464‐471. 25234743 10.12659/AOT.890893

[5] Orons PD , Zajko AB . Angiography and interventional aspects of renal transplantation. Radiol Clin North Am. 1995;33(3):461‐471. 7740106

[6] Jordan ML , Cook GT , Cardella CJ . Ten years of experience with vascular complications in renal transplantation. J Urol. 1982;128(4):689‐692. 6754971 10.1016/s0022-5347(17)53136-5

[7] Brown ED , Chen MY , Wolfman NT , Ott DJ , Watson NE Jr . Complications of renal transplantation: evaluation with US and radionuclide imaging. Radiographics. 2000;20(3):607‐622. 10835115 10.1148/radiographics.20.3.g00ma14607

[8] Khankan AA , Maeda M , Osuga K , Murakami T , Nakamura H . Post‐kidney transplantation iliac artery stenosis due to iatrogenic injury: case report. Cardiovasc Intervent Radiol. 2003;26(2):186‐188. 12616412 10.1007/s00270-002-0471-x

[9] Ozban M , Aydin C , Dursun B , Yagci B , Birsen O , Tekin K . Post‐kidney transplantation external iliac artery stenosis due to vascular clamp: report of a case. J Vasc Bras. 2014;13(3):254‐256.

[10] Kelsoe JR , Greenwood TA , Akiskal HS , Akiskal KK . The genetic basis of affective temperament and the bipolar spectrum. Int Clin Psychopharmacol. 2012;28:e5‐e6.

[11] Hedegard W , Saad WEA , Davies MG . Management of vascular and nonvascular complications after renal transplantation. Tech Vasc Interv Radiol [Internet]. 2009;12(4):240‐262. doi:10.1053/j.tvir.2009.09.006 20005481

[12] Rossi AP , Vella JP . Hypertension, living kidney donors, and transplantation: where are we today? Adv Chronic Kidney Dis [Internet]. 2015;22(2):154‐164. doi:10.1053/j.ackd.2015.01.002 25704353

[13] Tian X , Ji B , Niu X , et al. Efficacy and safety of low‐dose aspirin on preventing transplant renal artery stenosis: a prospective randomized controlled trial. Chin Med J. 2023;136(5):541‐549. 36914946 10.1097/CM9.0000000000002574 PMC10106233

[14] Hurst FP , Abbott KC , Neff RT , et al. Incidence, predictors and outcomes of transplant renal artery stenosis after kidney transplantation: analysis of usrds. Am J Nephrol. 2009;30(5):459‐467. 19776559 10.1159/000242431

[15] Skræddergaard A , Nyvad J , Christensen KL , Hørlyck A , Mafi HM , Reinhard M . Difficulty and importance of diagnosing stenosis of renal branch artery in fibromuscular dysplasia: a case report. Blood Press [Internet]. 2021;30(6):416‐420. 34697979 10.1080/08037051.2021.1993735

[16] DeVries BL , Wechsler B , Yim D . Case report of transplant renal artery stenosis secondary to mechanical renal artery kinking: balloon angioplasty as a supportive diagnostic tool? Int J Surg Case Rep [Internet]. 2021;83:106052. 34098190 10.1016/j.ijscr.2021.106052 PMC8187818

[17] Safdar O , Alaifan F , Alshammakh S , Hakami M , Alghaithi DF . Diagnostically challenging case of renal artery stenosis in a pediatric patient. Cureus. 2020;12(1):1‐6. 10.7759/cureus.6538 PMC6939964 31929955

